# Defining the Binding Region in Factor H to Develop a Therapeutic Factor H-Fc Fusion Protein against Non-Typeable *Haemophilus influenzae*

**DOI:** 10.3389/fcimb.2016.00040

**Published:** 2016-04-13

**Authors:** Sandy M. Wong, Jutamas Shaughnessy, Sanjay Ram, Brian J. Akerley

**Affiliations:** ^1^Department of Microbiology and Immunology, University of Mississippi Medical CenterJackson, MS, USA; ^2^Division of Infectious Diseases and Immunology, University of Massachusetts Medical SchoolWorcester, MA, USA

**Keywords:** factor H, non-typeable *Haemophilus influenzae*, complement, P5, outer-membrane protein, lung infection

## Abstract

Non-typeable *Haemophilus influenzae* (NTHi) cause a range of illnesses including otitis media, sinusitis, and exacerbation of chronic obstructive pulmonary disease, infections that contribute to the problem of antibiotic resistance and are themselves often intractable to standard antibiotic treatment regimens. We investigated a strategy to exploit binding of the complement inhibitor Factor H (FH) to NTHi as a functional target for an immunotherapeutic containing the NTHi binding domain of FH fused to the Fc domain of IgG1. Chimeric proteins containing the regions that most FH-binding bacteria use to engage human FH, domains 6 and 7 (FH6,7/Fc) and/or 18 through 20 (FH18–20/Fc), were evaluated for binding to NTHi. FH6,7/Fc bound strongly to each of seven NTHi clinical isolates tested and efficiently promoted complement-mediated killing by normal human serum. FH18–20/Fc bound weakly to three of the strains but did not promote complement dependent killing. Outer-membrane protein P5 has been implicated in FH binding by NTHi, and FH6,7/Fc binding was greatly diminished in five of seven P5 deficient isogenic mutant strains tested, implicating an alternative FH binding protein in some strains. Binding of FH18–20/Fc was decreased in the P5 mutant of one strain. A murine model was used to evaluate potential therapeutic application of FH6,7/Fc. FH6,7/Fc efficiently promoted binding of C3 to NTHi exposed to mouse serum, and intranasal delivery of FH6,7/Fc resulted in significantly enhanced clearance of NTHi from the lung. Moreover, a P5 deficient mutant was attenuated for survival in the lung model, suggesting that escape mutants lacking P5 would be less likely to replace strains susceptible to FH6,7/Fc. These results provide evidence for the potential utility of FH6,7/Fc as a therapeutic against NTHi lung infection. FH binding is a common property of many respiratory tract pathogens and FH/Fc chimeras may represent promising alternative or adjunctive therapeutics against such infections, which are often polymicrobial.

## Introduction

Non-typeable *Haemophilus influenzae* (NTHi), a common cause of respiratory tract infections, is associated with otitis media and sinusitis in children, and exacerbations of chronic obstructive lung disease (COPD; Murphy et al., 2009; Sethi et al., 2016). NTHi is consistently found in the lower respiratory tract in 30% of COPD cases and recurrent infection by diverse NTHi strains results in exacerbation of this disease (Murphy and Sethi, 2002), which afflicts greater than 6% of adults and has been ranked the third leading cause of death in the U.S. (Centers for Disease Control and Prevention [CDC], 2012). Nasopharyngeal colonization with NTHi in infants predisposes to recurrent otitis media (Harabuchi et al., 1994) in which NTHi has recently emerged as the most frequent bacterial isolate (Kaur et al., 2013). NTHi can also cause invasive infections including bacteremia, pneumonia, and meningitis, especially in neonates and individuals that are immunocompromised or have comorbidities (Van Eldere et al., 2014; Collins et al., 2016). Otitis media is the leading cause of pediatric antibiotic prescription, with β-lactams representing the frontline therapeutics (McCaig et al., 2002; Grijalva et al., 2009). The spread of β-lactamase producing NTHi as well as β-lactamase-negative ampicillin resistant strains globally has led to use of broader spectrum agents with their attendant complications (Van Eldere et al., 2014). Whereas, vaccination has been effective against type b *H. influenzae* with implementation of the capsular conjugate vaccine (Ladhani, 2012), it is complicated in NTHi, which lack capsule and exhibit extensive antigenic diversity of immunogenic outer-membrane proteins among strains (Gilsdorf, 1998). The highly conserved NTHi protein D has been included in the pneumococcal PhiD-CV (Synflorix; GSK) vaccine, which has shown moderate efficacy against otitis media in clinical studies. However, PhiD-CV has not been evaluated for other conditions such as exacerbation of COPD. Moreover, a recent study in a murine lung model was unable to demonstrate protection against NTHi after immunization with PhiD-CV (Siggins et al., 2015). New non-antibiotic anti-infectives active against NTHi would be beneficial as primary or adjunctive therapies.

To survive in their mammalian hosts, pathogens possess multiple countermeasures against innate immune defenses, in which the complement system plays a major role (Ram et al., 2010). One strategy shared by NTHi and many medically important microbes is to bind to human complement inhibitors, including Factor H (FH), vitronectin, and C4b-binding protein, to dampen complement activation on their surfaces (Würzner, 1999; Kraiczy and Würzner, 2006; Blom et al., 2009). FH inhibits the alternative pathway of complement by serving as a cofactor for the factor I-mediated cleavage of C3b to the hemolytically inactive iC3b fragment (Pangburn et al., 1977). FH also causes “decay acceleration,” whereby it irreversibly dissociates the Bb fragment from the alternative pathway C3 convertase, C3bBb (Weiler et al., 1976; Whaley and Ruddy, 1976; Fearon and Austen, 1977). FH comprises 20 domains, also known as short consensus repeat domains (SCRs) or complement control protein domains (CCPs) that are arranged in the form of a single chain (Ripoche et al., 1988). The first four N-terminal domains are necessary and sufficient for complement inhibition (Sharma and Pangburn, 1996). Pathogens bind FH regions distinct from its complement inhibitory domains, information that has been used to engineer FH fusion proteins lacking complement-inhibitory activity to the Fc region of IgG and thereby exploit this virulence property as a therapeutic target to direct Fc mediated clearance of *Neisseria meningitidis* and *Neisseria gonorrhoeae* in animal models of infection (Shaughnessy et al., 2014, 2016). This approach would be attractive for treatment of infections with NTHi, which frequently cause disease in the context of co-infection by other pathogens (Broides et al., 2009), primarily involving *Streptococcus pneumoniae, Moraxella catarrhalis*, and *Streptococcus pyogenes*, each of which expresses FH binding proteins (Horstmann et al., 1988; Dave et al., 2001; Bernhard et al., 2014).

Previous studies have shown that NTHi bind to FH (Hallström et al., 2008), and that binding is mediated by the cell-surface outer-membrane protein P5, a member of the OmpA family of proteins, which contributes to resistance of NTHi to killing by complement (Langereis et al., 2014; Rosadini et al., 2014). P5 has also been described as an adhesin interacting with epithelial cells and mucosal surfaces via several potential binding partners including respiratory mucin (Reddy et al., 1996), Eustachian tube mucus (Miyamoto and Bakaletz, 1996), ICAM-1 (Avadhanula et al., 2006), and CEACAM-1 (Hill et al., 2001), however its role in interaction with CEACAM-1 was recently shown to be indirect (Tchoupa et al., 2015). In addition to virulence related phenotypes identified *in vitro*, P5 has been implicated in bacterial colonization in the chinchilla (Sirakova et al., 1994) and lung infection in mice (Wong et al., 2013; Euba et al., 2015). In this study we identify the regions in FH that bind to NTHi, determine the role of P5 in these interactions, and evaluate the ability of a fusion protein that combines the NTHi-binding fragment of FH with the Fc domain of IgG to mediate complement-dependent killing of NTHi and facilitate clearance of bacteria *in vivo* in a mouse lung model of NTHi infection.

## Materials and methods

### Media and *Haemophilus influenzae* growth conditions

NTHi clinical isolates NT127 (Wong et al., 2011), Hi375 (Mell et al., 2014), Hi486 (Hood et al., 1999), 86-028NP (Harrison et al., 2005), PittGG (Buchinsky et al., 2007), R2846 (Barenkamp and Leininger, 1992), and R2866 (Nizet et al., 1996; Erwin et al., 2005) were grown at 35 ± 1.5°C in Brain Heart Infusion supplemented with 10 μg/ml nicotinamide adenine dinucleotide (NAD) and 10 μg/ml hemin (sBHI) on agar plates or in sBHI broth. DNA was transformed into naturally competent *H. influenzae* prepared as described (Barcak et al., 1991). Gentamicin (Gm) and 3,4-cyclohexenoesculetin-β-D-galacto-pyranoside (S-gal, Sigma-Aldrich), and D-xylose were added to sBHI at 10 μg/ml, 300 mg/L, and 1 mM, respectively. FH/Fc binding, C3 deposition and serum bactericidal assays were performed on NTHi grown on chocolate agar plates as described below.

### Bacterial strains and mutants

ΔP5 deletion mutations in Hi375, Hi486, PittGG, 86-028NP, R2846, and R2866 were created by transformation of a ~3 kb PCR product amplified with primers 5 omp1 and 3 omp2 from *H. influenzae* Rd strain RP5G (Rosadini et al., 2014). The NT127 P5 deletion mutant complemented with the wildtype gene from NT127 at the *xyl* locus, NTP5X, or carrying empty vector sequences at the *xyl* locus, NTP5V, have been described previously (Rosadini et al., 2014). The allelic exchange PCR product for deletions contains a replacement of the P5 coding region with the *aacC1* gentamicin (Gm) resistance cassette and flanking regions for homologous recombination. Gm^R^ transformants were selected on sBHI agar containing Gm. All strain constructions were verified by PCR amplification across the inserted recombinant region with primers specific for flanking sequences not contained in sequences within the P5 knockout exchange delivery DNA and by PCR amplification to verify presence of the gentamicin resistance cassette with aacC1 5′ (ATGTTACGCAGCAGCAACGATGTTACGCAGCAGG) and 3′ (TTAGGTGGCGGTACTTGGGTCGAT) primers to the coding region. Strains were additionally verified via Coomassie Brilliant Blue staining of whole-cell lysates after SDS-PAGE, and all mutants were deficient in an ~37–39 kDa band present in the wild-type parents consistent with the predicted sizes of the corresponding P5 proteins (Figure S1).

### Complement

Human serum was obtained from normal healthy adult volunteers who provided informed consent. Participation was approved by the University of Massachusetts Institutional Review Board for the protection of human subjects. Serum was obtained by allowing blood to clot at 25°C for 30 min followed by centrifugation at 1500 g for 20 min at 4°C. To study the effects of the FH/Fc proteins without confounding by natural anti-NTHi antibodies present in NHS, we depleted IgG and IgM from freshly collected human serum, as described previously (Ray et al., 2011). Briefly, EDTA (final concentration 10 mM) and NaCl (final concentration 1 M) were added to freshly prepared human serum and treated sera was passed first over anti-human IgM agarose (Sigma), followed by passage through protein G-Sepharose; both columns were equilibrated in PBS containing 10 mM EDTA and 1 M NaCl. NaCl was added to minimize C1q depletion during passage of serum through the anti-human IgM column. The flow-through was collected, spin concentrated and dialyzed against PBS/0.1 mM EDTA to its original volume using a 10-kDa cutoff Amicon Ultra-15 centrifugal filter device (Millipore, Bedford, MA), sterilized by passage through a 0.22-μm filter (Millipore), aliquoted and stored at −70°C. Hemolytic activity was confirmed using a total complement hemolytic plate assay (The Binding Site Inc., Birmingham, U.K). Depletion of IgG and IgM was confirmed by dot-blot assays. In some experiments, complement activity of serum was destroyed by heating serum at 56°C for 1 h. Mouse complement was obtained by allowing blood obtained by terminal cardiac puncture to clot for 20 min at room temperature followed by incubation for 20 min on ice. Serum was collected after centrifugation at 10,000 g for 10 min at 4°C and stored in single-use aliquots at −80°C. This procedure was performed in accordance with approved IACUC protocols at the University of Massachusetts Medical School.

### FH/Fc fusion proteins

Cloning, expression and purification of a chimeric protein comprising human FH domains 18–20 fused to human IgG1 Fc (FH18–20/Fc) and FH domains 6 and 7 fused to human IgG1 Fc (FH6,7/Fc) have been described previously (Shaughnessy et al., 2011, 2014). Plasmids encoding the FH/Fc fusion proteins were used to transiently transfect CHO cells using lipofectin (Life Technologies), according to the manufacturer's instructions. Media from transfected cells was collected after 2 days and FH/Fc was purified by passage over protein A agarose. Mass was determined by Coomassie Blue staining of proteins separated by SDS-PAGE and protein concentrations were determined using the BCA protein Assay kit (Pierce).

### Antibodies

Anti-human IgG FITC (Sigma), anti-human C3c-FITC (BioRad), and anti-mouse C3 FITC (MP Biomedicals) were used in flow cytometry assays, all at a dilution of 1:100 in Hanks Balanced Salt Solution (HBSS) containing 0.1% BSA and 1 mM CaCl_2_ and 1 mM MgCl_2_ (HBSS^++^/BSA).

### Flow cytometry

Binding of FH/Fc fusion proteins to bacteria and human and mouse C3 deposition on NTHi were performed by flow cytometry as described previously (Shaughnessy et al., 2011, 2014; Rosadini et al., 2014). All incubations with proteins, serum and Ab were carried out in HBSS^++^/BSA. Data were acquired on a BD FACSCalibur flow cytometer and data were analyzed using FlowJo software.

### Serum bactericidal assays

NTHi harvested from chocolate agar plates following overnight growth were repassaged onto fresh chocolate agar plates and grown for 5 h at 37°C in an atmosphere containing 5% CO_2_. Bacteria were resuspended in HBSS^++^/BSA. The reaction mixture contained 20% human complement and ~1000 CFU of NTHi and the indicated concentrations of FH/Fc in a final volume of 75 μl. Aliquots of 12.5 μl reaction mixtures were plated onto chocolate agar in duplicate at the beginning of the assay (t_0_) and again after incubation at 37°C for 30 min (t_30_). Survival was calculated as the number of viable colonies at t_30_ relative to t_0_.

### *H. influenzae* competition and FH protection assays in the murine lung model

*H. influenzae* grown to mid-log phase (OD_600_ 0.3–0.5) in 5 ml sBHI were pelleted, resuspended at the appropriate concentrations in Hank's balance salt solution containing calcium and magnesium chloride, and inoculated (40 μl) intranasally into the nares of 6–8 week-old female C57BL/6 mice (Charles River Laboratories, Wilmington, MA) anesthetized with ketamine (50 mg/kg) and xylazine (5 mg/kg) by intraperitoneal (IP) injection. Lungs were harvested and homogenized in 2 ml of BHI at 20 h post-bacterial inoculation and plated onto sBHI agar (with S-gal and D-xylose for the *in vivo* competition assay experiments) and grown for colony forming unit (CFU) determination.

For *in vivo* competition assays to assess virulence of the ΔP5 mutant relative to virulence of the wild-type, 10^7^ CFU of NTHi NT127 containing empty cloning vector, NTV, (parent), Δ*P5* mutant containing empty vector, NTP5V (Δ*P5)*, and Δ*P5* mutant with complementing copy of *P5 in trans*, NTP5X (*P5* complemented; Rosadini et al., 2014) were co-inoculated intranasally with an equal number of an *H. influenzae* LacZ+ expressing competitor strain (Rosadini et al., 2011) into the nares of mice (*n* = 5). Ratio of CFU of the experimental strain (white colonies, LacZ−) to competitor strain (black colonies, LacZ+) was reported as the competitive index.

For protection assays with FH6,7 human IgG1 Fc fusion protein (FH6,7/HuFc; Shaughnessy et al., 2014) 10^7^ CFU NT127 wild-type was co-inoculated with 50 μg of protein intranasally into mice (*n* = 10 for FH6,7/HuFc group; 8 for PBS control group), and bacterial CFU recovered from lungs as above. Experiments were conducted with approval and in accordance with guidelines of the Institutional Animal Care and Use Committee at the University of Mississippi Medical Center (Jackson, MS).

## Results

### P5 on NTHi NT127 interacts with FH domains 6 and 7

**Figure 1:**
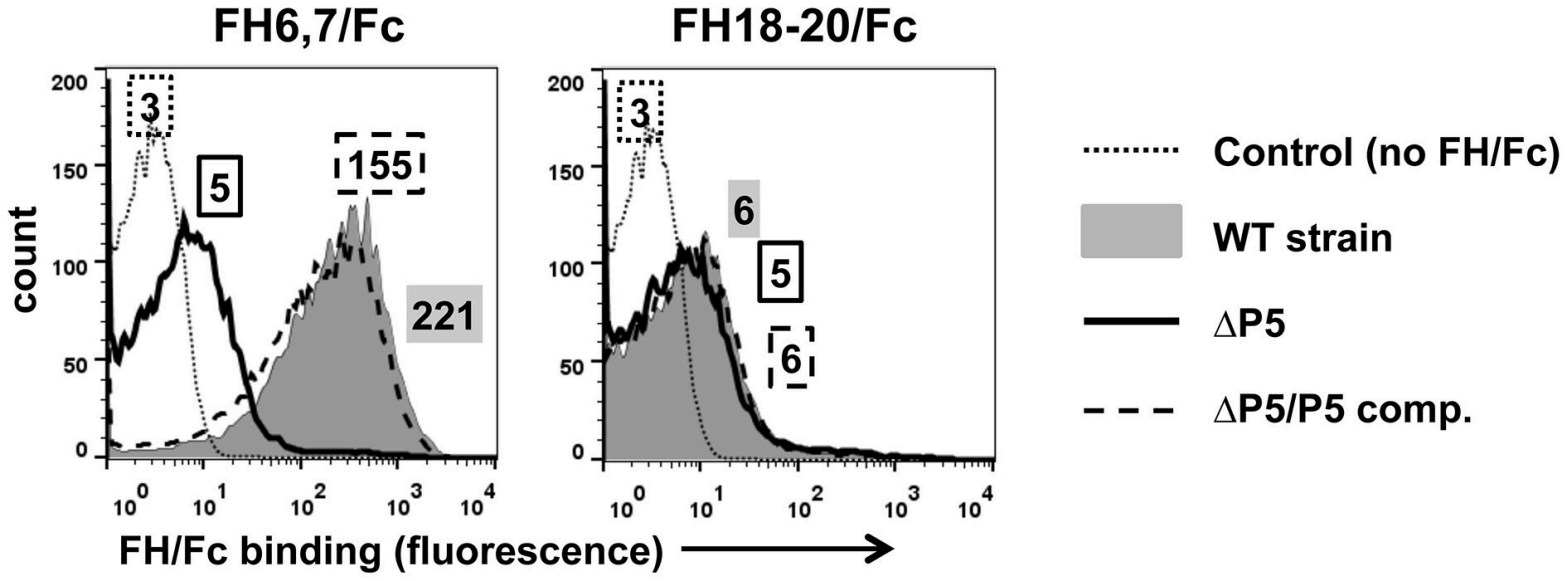
**NTHi NT127 P5 binds FH domains 6 and 7**. NT127, P5 deletion mutant NTP5V, (ΔP5), and the complemented P5 deletion mutant NTP5X, (ΔP5/comp) were incubated with 10 μg/ml of FH6,7/Fc (left graph), or FH18–20/Fc (right graph) and bound FH/Fc was detected with anti-human IgG FITC. “Control” represents the wild-type strain incubated with anti-human IgG FITC (no added FH/Fc). Numbers alongside the histograms indicate the median fluorescence intensity of the entire bacterial population; the outline/shading of each value corresponds to that used for the histogram. X-axis, fluorescence on a log_10_ scale; Y-axis, counts. One representative experiment of two reproducible repeats is shown.

Most microbes that bind human FH interact with a region spanned by FH domains 6 and 7 and/or 18 through 20. We therefore examined binding of two FH/Fc fusion proteins, FH6,7/Fc and FH18–20/Fc that spanned these common microbial binding regions in FH, to NTHi strain NT127. To verify that P5 was indeed the molecule that interacted with the FH fragments, we compared wild-type to a P5 deletion mutant (ΔP5) and a complemented strain (ΔP5/comp). As shown in Figure 1, the wild-type strain bound FH6,7/Fc strongly, but barely bound FH18–20/Fc. Loss of P5 almost totally abrogated binding of FH6,7/Fc, which was restored with complementation. A dose response binding assay revealed that near maximal binding of FH6,7/Fc was achieved at 20 μg/ml (Figure S2). These data suggest that P5 on NT127 interacts with FH domains 6 and 7.

### FH6,7/Fc enhances complement deposition on and killing of NTHi NT127

**Figure 2:**
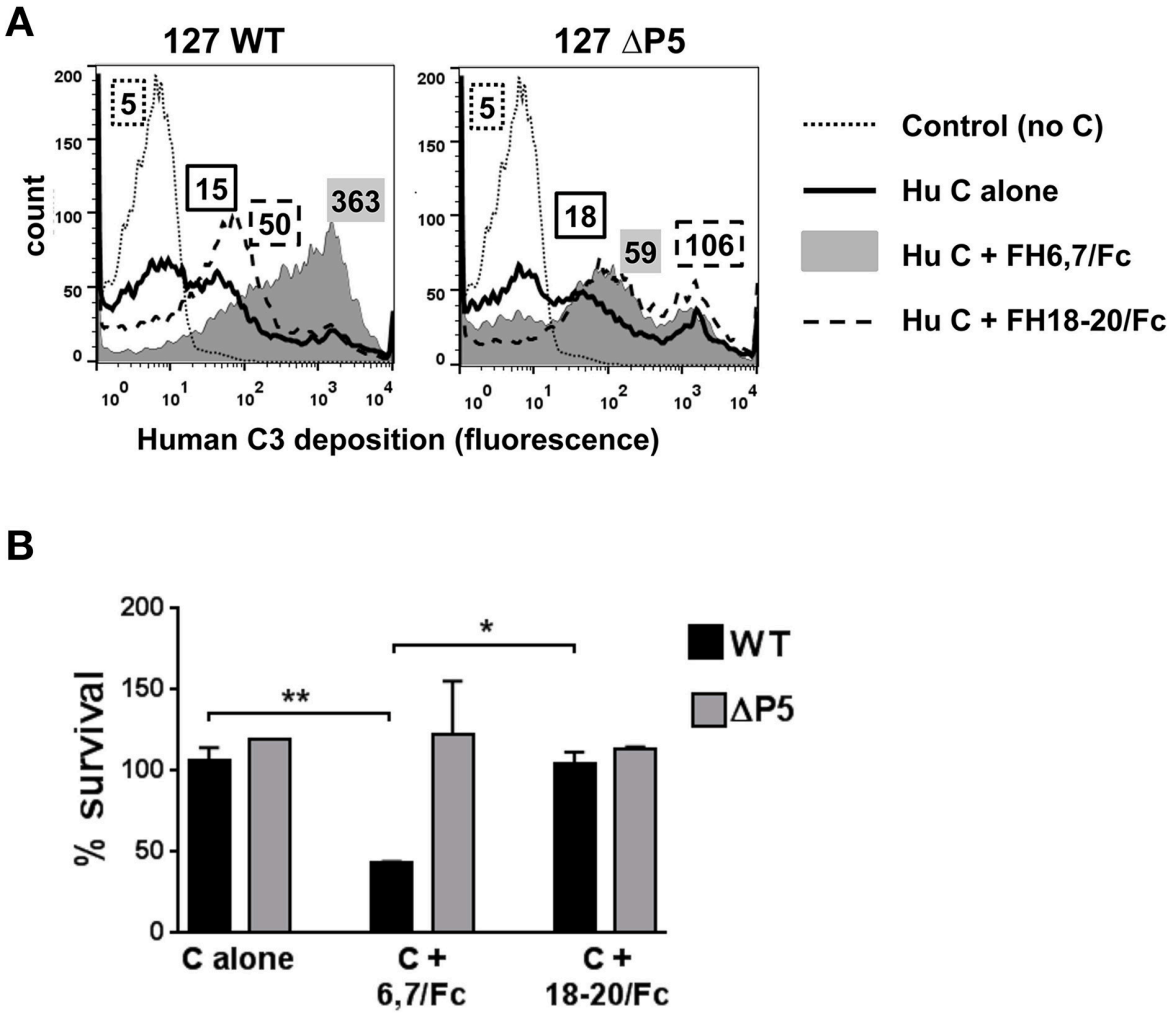
**FH6,7/human Fc mediates human C3 deposition and complement-dependent killing of NTHi NT127**. **(A)** FH6,7/Fc enhances C3 deposition on NT127. Wild-type strain NT127 (127 WT; left graph) and 127 ΔP5 (right graph) were incubated with human complement (C) alone, or C plus either FH6,7/Fc or FH18–20/Fc, each at a concentration of 20 μg/ml. Following incubation at 37°C for 30 min, C3 deposited on bacteria was measured by flow cytometry. “Control” represents the wild-type strain incubated in buffer alone (no C added). X-axis; fluorescence on a log_10_ scale; Y-axis, counts. Numbers alongside the histograms indicate the median fluorescence intensity of the entire bacterial population; the outline/shading of each value corresponds to that used for the histogram. One representative experiment of two reproducible repeats is shown. **(B)** FH6,7/Fc enhances C-dependent killing of NTHi NT127. Strain NT127 and its ΔP5 mutant were incubated with 20% C alone, or 20% C plus either FH6,7/Fc or FH18–20/Fc each at a final concentration of 20 μg/ml, and percent survival of bacteria (shown on the Y-axis) was measured in a serum bactericidal assay. Each bar represents the mean (range) of two separate experiment. ANOVA was used to compare the survival of each strain under the three incubation conditions. ^*^P < 0.05; ^**^P < 0.01.

Because several medically important microbes bind to similar regions in FH, FH fragments fused to Fc have the potential to serve as anti-bacterial immunotherapeutics. We asked whether FH6,7/Fc could activate complement on NTHi NT127. As shown in Figure 2A, FH6,7/Fc enhanced C3 fragment deposition on the wild-type strain (gray histogram in the left panel) relative to C3 deposited on bacteria incubated either with complement alone (solid line) or complement plus the control protein FH18–20/Fc that bound only weakly to strain NT127 (dashed line). Compared to the wild-type strain, the P5 deletion mutant showed a smaller increase in C3 deposition in the presence of the FH/Fc's and the C3 deposition histograms in the presence of both FH/Fc molecules were similar.

To determine whether FH6,7/Fc could affect killing of NT127, bacteria were incubated with either complement alone, or complement plus each of the FH/Fc's (each at 20 μg/ml). Exposure to FH6,7/Fc in the presence of complement resulted in killing of the wild-type strain (Figure 2B), whereas survival of the ΔP5 mutant was not affected under this condition. No killing of either strain was observed with complement alone, or complement plus FH18–20/Fc.

### FH6,7/Fc mediates killing of six additional clinical NTHi isolates

**Figure 3:**
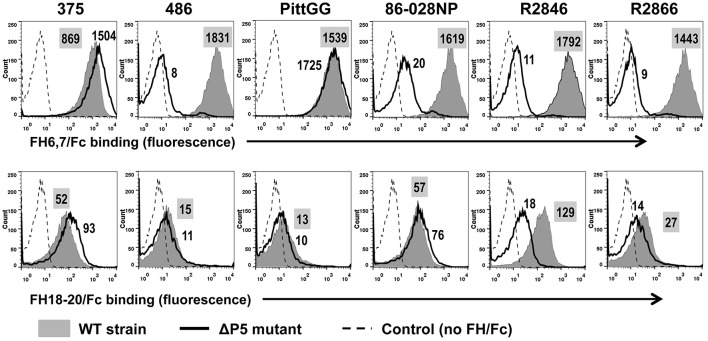
**Binding of FH6,7/Fc (upper panel) and FH18–20/Fc (lower panel) to six additional NTHi isolates and their P5 deletion mutants (ΔP5)**. Flow cytometry to measure the amount of binding of the two FH/Fc fusion molecules was carried out as described in Figure 1. Axes are as described in Figure 1. Numbers alongside the histograms indicate the median fluorescence intensity of the entire bacterial population; the number in the shaded box represents binding to the wild-type strain. Control reactions represent wild-type bacteria incubated with anti-human IgG FITC (no FH/Fc added). The median fluorescence for the controls was ~5 and has not been indicated in the figure for simplicity.

To ascertain that binding and activity of FH6,7/Fc was not restricted to a single NTHi isolate, we studied six additional NTHi strains and their ΔP5 mutants. All wild-type NTHi strains bound FH6,7/Fc (Figure 3; gray shaded histograms in the upper panel). Rather surprisingly, the P5 deletion mutants of two isolates, 375 and PittG, continued to bind FH6,7/Fc, suggesting that these strains could bind to FH domains 6 and 7 through a molecule distinct from P5. It is worth noting that the P5 deletion mutant of 375 showed an increase in FH6,7/Fc binding compared to the parent strain (1.7- and 1.4-fold increase in fluorescence in two experiments performed).

Binding of FH18–20/Fc showed varying patterns. Similar to findings with strain NT127, strains 486 and PittGG bound minimal amounts of FH18–20/Fc. The four other strains (375, 86-028NP, R2846, and R2866) bound FH18–20/Fc to varying degrees, with maximum binding seen to strain R2846 and the lowest binding seen with R2866. However, the amount of binding noted was ~10- to ~50-fold less than the corresponding FH6,7/Fc binding. Deleting P5 decreased FH18–20/Fc binding to R2846, suggesting that P5 expressed by this strain could interact with the C-terminus of FH. As observed with FH6,7/Fc, a small increase in FH18–20/Fc binding to the ΔP5 mutant of 375 was detected.

**Figure 4:**
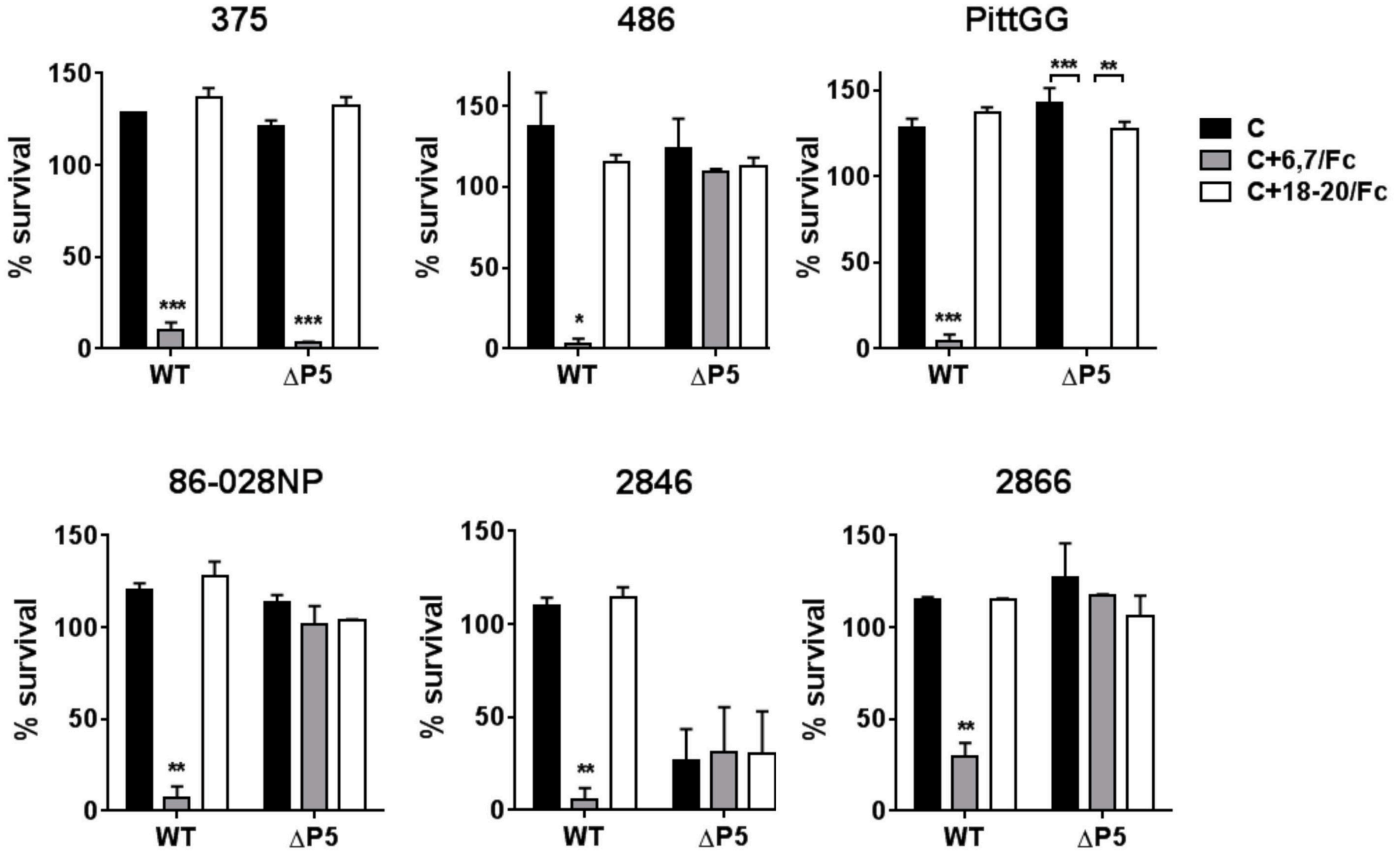
**Bactericidal activity of FH6,7/Fc against NTHi and their P5 deletion mutants**. Serum bactericidal assays were performed using 20% human complement either alone (C) or in the presence 10 μg/ml of FH6,7/Fc or FH18–20/Fc. Survival of bacteria at 30 min relative to baseline CFU at the start of the assay is shown on the Y-axis. The mean (range) of two separate experiments is shown. Comparison of survival across the three reaction conditions for each strain was made by ANOVA. ^*^P < 0.05;^**^P < 0.01; ^***^P < 0.001.

The ability of the FH/Fc's to mediate complement-dependent killing of the NTHi isolates was next examined. As shown in Figure 4, all six wild-type isolates showed < 50% survival in the presence of FH6,7/Fc and complement. Consistent with their ability to bind high amounts of FH6,7/Fc, the ΔP5 mutants of 375 and PittGG were also killed. None of the isolates were killed by FH18–20/Fc. Of note, the P5 deletion mutant of 2846 showed >50% killing with complement alone, and addition of either FH/Fc molecule had no further effect on survival.

Taken together, the data suggest that FH6,7/Fc binds to all NTHi strains tested. In some strains, FH domains 6 and 7 interact with a molecule on NTHi distinct from P5. FH6,7/Fc can mediate complement-dependent killing of all tested strains.

### Activity of FH6,7/Fc in a mouse model of NTHi lung infection

**Figure 5:**
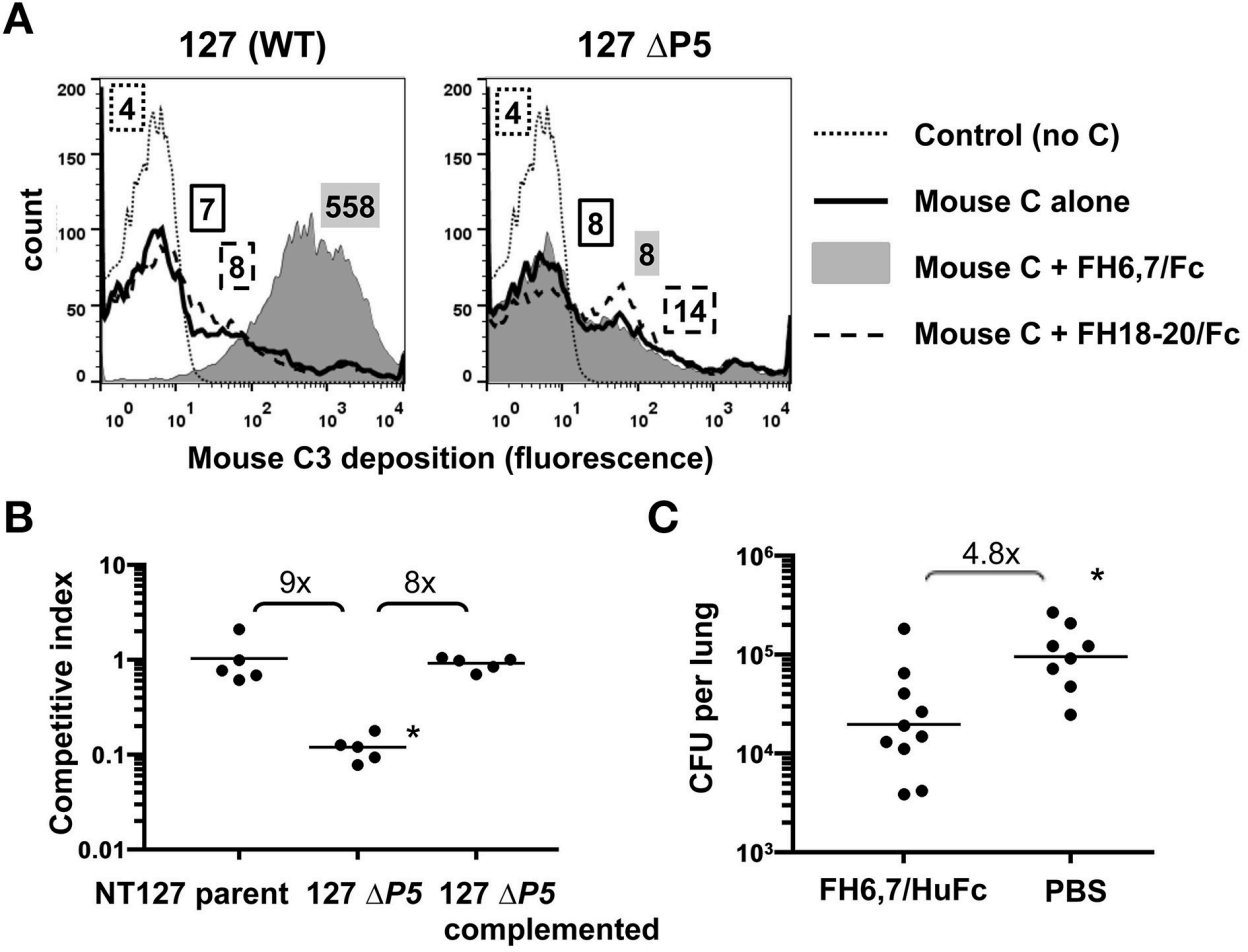
**FH6,7/Fc mediates mouse C3 deposition on NTHi, targets a structure required by NTHi during lung infection, and can decrease burden of NTHi in the murine lung model**. **(A)** FH6,7/Fc increases mouse C3 deposition on NTHI NT127. Strain NT127 and its ΔP5 mutant were incubated with mouse complement (Mouse C) alone, or mouse complement in the presence of 10 μg/ml of either FH6,7/Fc or FH18–20/Fc for 30 min at 37°C. Control reactions show the wild-type strain incubated in buffer alone followed by anti-mouse C3 FITC. Mouse C3 deposited on bacteria was measured by flow cytometry. Axes are as described in Figure 1. Numbers alongside the histograms represent the median fluorescence of the entire bacterial population and the shading/patterns associated with the numbers correspond to that used for the histogram. One representative experiment of two reproducible repeats is shown. **(B)** Loss of the FH6,7/Fc target protein P5 results in attenuation of NTHi survival in the mouse lung. Strain NT127, its ΔP5 mutant, and the ΔP5 complemented with wild-type P5 were each co-inoculated intranasally with an equal number of a *lacZ* expressing NT127 derived reference strain into C57BL/6 mice. Lungs were harvested 20 h post-inoculation for determination of bacterial CFU. Ratio of CFU recovered of the experimental strain (LacZ−) to competitor strain (LacZ+) is reported as the competitive index. Bars indicate means. Differences were statistically significant between the ΔP5 vs. the parent and complemented strains (^*^p < 0.01, ANOVA with Bonferroni's multiple comparison test). **(C)** FH6,7/Fc decreases burden of NTHi in the lung model. 10^7^ CFU NTHi NT127 and 50 μg of FH6,7/HuFc protein (control animals received PBS) were co-inoculated intranasally into C57BL/6 mice and lung CFU determined at 20 h post-inoculation. Bars, geometric means. Lower limit of detection, 200 CFU. Statistical analysis was by Mann-Whitney. ^*^P = 0.006.

As a prelude to testing the efficacy of FH6,7/Fc *in vivo*, its ability to deposit mouse complement on NTHi was tested. As shown in Figure 5A, FH6,7/Fc was capable of mediating mouse C3 deposition (gray shaded histogram in the left graph) consistent with results with human complement (Figure 1). Similarly, deposition of mouse C3 mediated by FH6,7/Fc was abrogated in the ΔP5 mutant, and that mediated by FH18–20/Fc slightly increased.

While P5 has been implicated in virulence in the chinchilla middle ear (Sirakova et al., 1994), it is possible that it is not required for bacterial survival in the lung, which would provide a means of escape from a FH6,7/Fc based therapeutic by strains that lose P5 via mutation. Therefore, we evaluated the ΔP5 mutant in the lung model. As shown in Figure 5B, survival of the ΔP5 mutant was significantly attenuated, and complementation restored survival that of the parent strain.

To evaluate the ability of FH6,7/Fc to reduce the burden of NTHi infection, we utilized the mouse lung model. FH6,7/Fc vs. PBS carrier alone was delivered intranasally concurrent with NTHi NT127 inoculation and lungs harvested for bacterial CFU determination after 20 h. The mean CFU of NTHi recovered from the lungs of FH6,7/Fc treated mice was 4.8-fold lower than that of NTHi infected mice receiving carrier alone, and this difference was statistically significant (Figure 5C).

## Discussion

In this report, we have defined domains 6 and 7 as a region in FH that binds to all seven strains of NTHi studied. Five of the seven strains bound FH domains 6 and 7 through P5. Deleting P5 from two strains (375 and PittGG) did not decrease FH6,7/Fc binding, suggesting that these strains bound FH through an alternate molecule(s). The presence of a molecule on NTHi distinct from P5 that bound FH was also suggested by Langereis et al. (2014), who observed residual FH binding to the P5 deletion mutants of two of the three NTHi strains that they evaluated. FH domains 18 through 20 also interacted with strains 375, 86-028NP, R2846 and to a lesser extent, strain R2866. P5 of strain R2846 bound to FH domains 18–20, evidenced by the decrease in binding noted upon deleting P5. Residual FH18–20/Fc binding to R2846 ΔP5 suggests the presence of an alternate FH ligand on this strain. Unexpectedly, deletion of P5 in strain R2846 resulted in sensitivity to complement even in the absence of FH/Fc fusion proteins. It is possible that the R2846 ΔP5 mutant is more susceptible, relative to other strains, either to the alternative and/or lectin complement pathways. Another possibility is that R2846 ΔP5 may be hypersensitive to residual IgM in antibody depleted human serum; we showed previously that P5 expression may facilitate evasion of the classical pathway by decreasing binding of IgM to the bacterial surface (Rosadini et al., 2014). Strain 86-028NP also showed evidence for two distinct molecules that bound to FH; similar to R2846, the alternate ligand was implicated in interacting with FH domains 18–20. Similar to the two binding regions in FH observed for NTHi in this study, Hallstrom and colleagues showed previously that *H. influenzae* type b (Hib) strains also interacted with FH via a region spanned by domains 6 and 7 and 18–20 (Hallström et al., 2008). Meri et al further confirmed binding of the two C-terminal domains of FH to *H. influenzae* (Meri et al., 2013). A ligand for FH on Hib was subsequently identified by Fleury et al. as a lipoprotein that was named protein H (PH; encoded by a gene called *lph*), which is not present in NTHi strains (Fleury et al., 2014).

An interesting observation was the increase in binding of both FH6,7/Fc and FH18–20/Fc upon deleting P5 from strain 375. Possible explanations for this observation include increased expression of the alternate ligand for FH and/or increased exposure of the FH binding regions of the alternate binding site(s) when P5 is deleted. Collectively, these data suggest that the interactions between the two major microbial binding regions in FH and NTHi strains are complex and a summary of the binding data between the FH/Fc's and the strains of NTHi used in this study is provided in Table 1.

**Table 1:** **Summary of interactions between FH and NTHi**.

**NTHi strain**	**FH ligand(s)**	**Binding region(s) in FH**
NT127	P5	6,7
375	?	6,7; 18–20
486	P5	6,7
PittGG	?	6,7
86-028NP	P5	6,7
	?	18–20
R2846	P5	6,7
	?	18–20
R2866	P5	6,7

We have shown previously that FH/Fc fusion molecules activate complement on the surface of *N. meningitidis* and *N. gonorrhoeae*. FH6,7/Fc decreased the burden of meningococcal bacteremia in infant Wistar rats (Shaughnessy et al., 2014) and a derivative of FH18–20/Fc that contained a D → G mutation at position 1119 in domain 19 decreased the burden of gonococcal vaginal colonization in BALB/c mice (Shaughnessy et al., 2016). Encouraged by these findings, we explored the ability of the FH/Fc fusion proteins to mediate complement-dependent killing of NTHi. Each of the seven NTHi strains tested were killed (< 50% survival) by FH6,7/Fc plus complement. The P5 deletion mutants of 375 and PittG that also bound FH6,7/Fc were also killed. Thus, targeting either P5 or the alternate receptor(s) by FH6,7/Fc results in complement-dependent killing. It is worth noting that none of the strains showed killing over baseline levels (complement alone) when FH18–20/Fc was present in the reaction mixture. Lack of bactericidal activity of FH18–20/Fc is likely because the density of Fc achieved on the bacterial surface does not reach the threshold required to engage sufficient C1 complex to trigger C4 activation and subsequent downstream C3 deposition and membrane attack complex formation.

The C-terminus of FH plays an important role in regulation of complement activation on host cell surfaces. The interactions of domains 19 and 20 of FH interact with C3b fragments and select glycosaminoglycans, respectively, rendering the cell surface a complement non-activator (Kajander et al., 2011; Blaum et al., 2015). The interaction of FH with sialic acid increases its affinity for C3b and promotes the cofactor and decay accelerating activities of FH (Fearon, 1978; Kazatchkine et al., 1979; Meri and Pangburn, 1990). To overcome the potential concern of FH18–20/Fc interfering with the function of endogenous FH, we introduced a D → G mutation in domain 19 of FH18–20/Fc, which abrogated hemolysis of anti-CD59-treated human RBC by autologous serum (Shaughnessy et al., 2016). Although not implicated in limiting complement activation on normal host cells, FH domain 7 may play a critical role in certain pathological conditions. Individuals with the 402H polymorphism in FH domain 7 are at a higher risk for developing age-related macular degeneration than persons with Y at position 402 (Haines et al., 2005; Klein et al., 2005). Weismann and colleagues showed that the 402H polymorphism decreased the affinity of FH for malondialdehyde, a lipid peroxidation product that accumulates in lesions of AMD called drusen (Weismann et al., 2011), thereby enabling greater alternative pathway activation and hastening ocular damage. We acknowledge that the development of FH/Fc molecules as therapeutics should proceed with caution so as not to interfere with the physiologic functions of FH. Further, studies on the pharmacokinetics and stability of FH/Fc *in vivo* are also important considerations in drug development.

P5 has been considered as a vaccine candidate against NTHi, however the range of available immunogenic yet conserved epitopes of this protein is limited by its antigenic heterogeneity between strains. The approach of targeting a functional interaction involved in pathogenesis may aid in bypassing this diversity. P5 may be an attractive target for such novel therapeutics because this molecule is ubiquitously expressed, is not phase-variable, and is not subject to epigenetic regulation (Atack et al., 2015). Further, P5 deletion mutants show reduced virulence in the chinchilla otitis media model and mouse lung model compared to their wild-type counterparts (Sirakova et al., 1994), which we have substantiated in the mouse lung model with isogenic strains and complementation of the mutant by restoration of wildtype P5 at an ectopic chromosomal location (Figure 5B). Resistance to FH6,7/Fc, if this were to occur, would require selection of P5 deletion mutants or P5 mutants that lack the ability to bind to human FH, both of which may place the bacterium at a fitness disadvantage as indicated by attenuation of survival of NTHi in the lung model conferred by deletion of P5. Alternatively, it is possible that mutational loss of FH binding by P5 may not strongly influence the course of infection if, for example, other P5 virulence phenotypes mediated by P5 exhibit predominant effects and do not require the same structural regions. Langeries et al. have identified the predicted surface exposed loops 1 and 2 in P5 of NTHi strain R2866 as the FH interacting regions (Langereis et al., 2014), and it is possible that the surface exposed loops 3 and 4 of P5 mediate alternative functions during infection. It will be of interest to determine whether the role of P5 proteins in virulence vs. FH binding can be dissociated. Identification of the alternate acceptor molecule(s) for FH6,7/Fc also merits further consideration.

As an initial test of the potential therapeutic utility of FH6,7/Fc against NTHi we evaluated its ability to decrease survival of NTHi in a mouse model. Murine C3 was efficiently targeted by FH6,7/Fc to the bacterial surface in the presence of mouse serum *in vitro*, similar to human C3 (Figure 5A), suggesting feasibility. Encouragingly, when delivered intranasally into the lung, FH6,7/Fc was able to reduce the burden of NTHi compared to mock treatment, producing a statistically significant 4.8-fold decrease in recovered bacteria 20 h after inoculation (Figure 5C). We note that this was a partial effect, and eradication of an ongoing infection will likely require repeated doses. In addition, clinical application would likely require aerosolization of the fusion protein to achieve similar exposure to the bacteria, however this delivery method is well-tolerated in patients treated for other conditions and is a clinically feasible approach. We have provided evidence for the efficacy of FH6,7/Fc *in vivo*, however, we acknowledge that further work will be needed to define its efficacy against NTHi infection in other niches such as the middle ear, sinuses, and the bloodstream. Further, only human but not mouse FH has been reported to bind to NTHi (Langereis et al., 2014). Thus, the efficacy of FH6,7/Fc *in vivo* in the presence of human complement inhibitors that may counteract the efficacy of the therapeutic merits study. Nevertheless, enhanced C3 deposition and killing of NTHi by FH6,7/Fc in human serum that contains endogenous FH provides optimism for activity of this molecule in the context of human complement.

In conclusion, FH6,7/Fc may prove a novel and promising adjunctive therapeutic against NTHi infections, particularly in instances of recurrent or recalcitrant infections, where multiple courses of antibiotics have proven ineffective or only partially effective and may be associated with adverse side effects.

## Author contributions

SW, JS, SR, and BA designed the study, performed experiments, analyzed data, and wrote the manuscript. JS and SW contributed equally.

### Conflict of interest statement

The authors declare that the research was conducted in the absence of any commercial or financial relationships that could be construed as a potential conflict of interest.
