# Supplementary figures and images for: Defining the Binding Region in Factor H to Develop a Therapeutic Factor H-Fc Fusion Protein against Non-Typeable Haemophilus influenzae
Source: Front Cell Infect Microbiol. 2016 Apr 13;6:40. doi: 10.3389/fcimb.2016.00040 (PMC4829610; doi:10.3389/fcimb.2016.00040)

Fig. S1

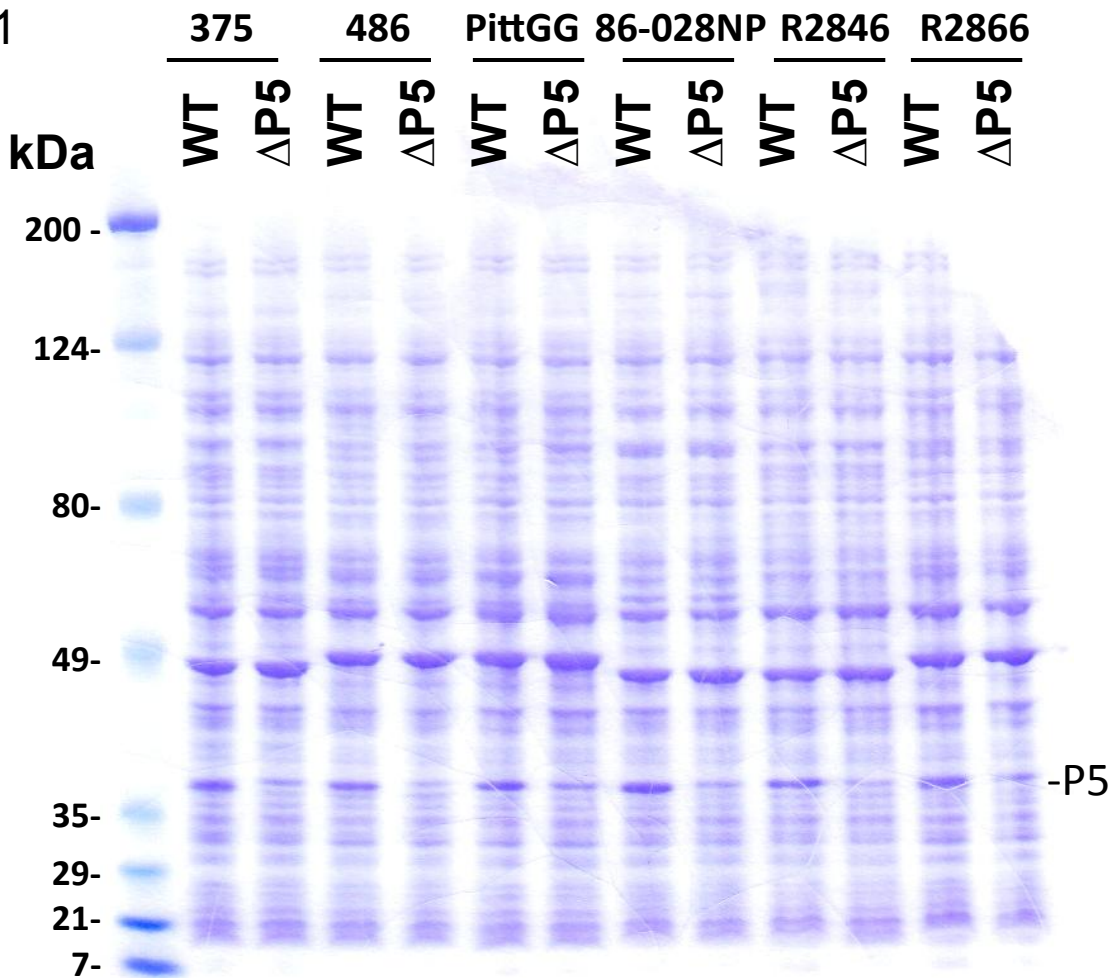

Fig. S2

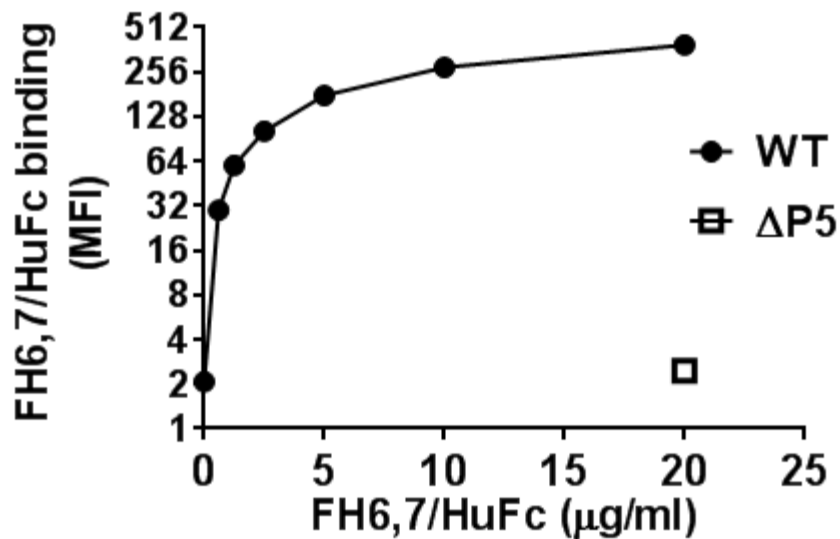

Supplement: Supplementary file 1 [file Image1.PDF]
